# Early Ectopic Pregnancy Refractory to Methotrexate Treatment: A Case Report

**DOI:** 10.7759/cureus.19686

**Published:** 2021-11-18

**Authors:** ChiaJu Lin, HsiaoYun Hsieh

**Affiliations:** 1 Department of Obstetrics, Gynecology & Women's Health, Taichung Veterans General Hospital, Taichung, TWN

**Keywords:** laparoscopic salpingostomy, serum beta hcg, ruptured tubal ectopic pregnancy, general gynecology, transvaginal ultrasonography, laparoscopic surgery, methotrexate, tubal ectopic pregnancy

## Abstract

Methotrexate (MTX) is known as a systemic treatment for early ectopic pregnancy with low serum beta-human chorionic gonadotropin (βhCG) levels. Here we present our experience of an unsatisfactory outcome following MTX treatment for early tubal pregnancy.

The case is a 39-year-old female with left tubal ectopic pregnancy and a history of one right tubal ectopic pregnancy and an uneventful episode of delivery. In the absence of any contraindications, the patient underwent initial MTX treatment. At first, her serum βhCG level was 1,258 mIU/mL but remained elevated. Then she underwent a second and third dose of MTX. After a month, the serum βhCG level had not declined to within an acceptable range. The ectopic mass was enlarged as determined by transvaginal ultrasonography and hemoperitoneum. A laparoscopic salpingectomy was performed.

Early ectopic tubal pregnancy can be managed medically with a high success rate. However, repeat ectopic pregnancy indicates an increased risk of treatment failure to medical treatment, and should be mentioned to the patient when discussing their treatment options.

## Introduction

Ectopic pregnancy (EP) is defined as implantation of the blastocyst outside the uterine endometrial cavity, and over 95% occur in the fallopian tube [1]. As a potentially life-threatening gynecological condition [2], its management is determined by the timing of the diagnosis and whether the patient is hemodynamically stable.

A diagnosis of tubal pregnancy is based on transvaginal ultrasonography (TVUS) and serum levels of beta-human chorionic gonadotropin (βhCG). If the intrauterine gestational sac has not been found by TVUS when serum βhCG reaches between 1,500 and 3,000 mIU/mL [3], a diagnosis is established. The slow elevation ratio of repeated βhCG, <53% in two days, could also indicate a non-viable intrauterine pregnancy [4,5].

Tubal EP can be managed surgically, medically, or expectantly. Methotrexate (MTX) is the medical treatment for early EP and other gynecological neoplasms. The treatment protocols include single and multiple doses. A single dose intramuscular injection of MTX at a dosage of 1 mg/kg or 50 mg/m^2^ is recommended as the first-line treatment protocol [6]. The success rate is >90% when the diagnosis is made when serum βhCG is <2,000 mIU/mL [7].

Factors that impact the efficacy of MTX treatment include a high baseline serum βhCG (>5,000 mIU/mL) [7,8], fetal cardiac activity on the TVUS, and large size of the ectopic mass (≥3.5 cm) [9]. However, there are still undetected factors that may have a negative impact on the treatment efficacy of MTX. In this case report, we share our experience of an unsatisfactory outcome following MTX treatment for early tubal pregnancy.

## Case presentation

A 39-year-old female (gravida 2, para 1) presented to the outpatient department in a tertiary referral hospital in Taiwan when her menstrual period was 10 days overdue on June 2021. She had a history of right tubal EP 10 years previously and had undergone MTX treatment once before. She then received a diagnostic laparoscopic surgery because of a right tubal occlusion. She had a spontaneous pregnancy and an uneventful vaginal delivery at full term in 2015. Otherwise, her medical history was relatively unremarkable and she had no known allergies. Her last menstrual period (LMP) was 43 days ago, in May 2021. On presentation, she started to have a little vaginal bleeding. Based on the date of her LMP, she was estimated to be at six weeks gestation.

**Figure 1:**
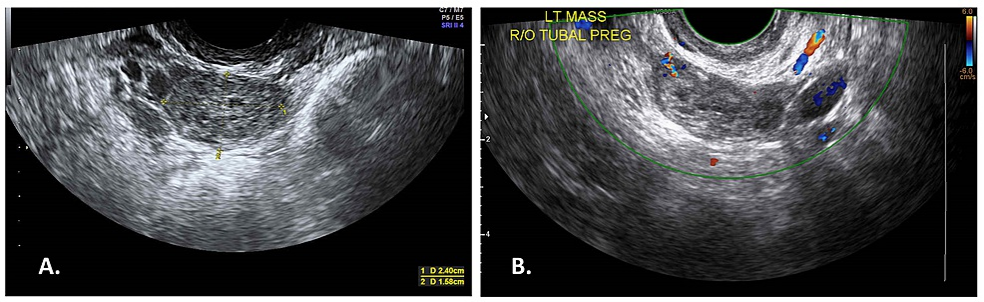
TVUS on the first OPD day and initial MTX day. A: TVUS on the first OPD day. A left tubal ectopic mass measuring 2.4 x 1.58 cm was observed. There was no yolk sac or fetal pole formation. B: TVUS on the day of initial MTX treatment (nine days after first OPD). The left tubal ectopic mass measured 2.3 x 1.3 cm. TVUS: transvaginal ultrasonography; OPD: outpatient department; LT: left. R/O: rule out. PREG: pregnancy.

A physical examination revealed her to be conscious and alert, vitally stable, and afebrile. Her abdomen was soft without tenderness. TVUS revealed an anteverted uterus measuring 8.8 x 4.8 cm in size, with an endometrial thickness of 7 mm and without a gestational sac in the endometrial cavity. A hyperechoic left adnexal mass measuring 2.4 x 1.58 cm in size was observed. There was no yolk sac or fetal pole formation (Figure 1A). Laboratory data reported that the patient’s βhCG level was 286.3 mIU/mL. We considered the condition of spontaneous tubal abortion or early-stage EP, and so we scheduled repeat tests.

**Figure 2:**
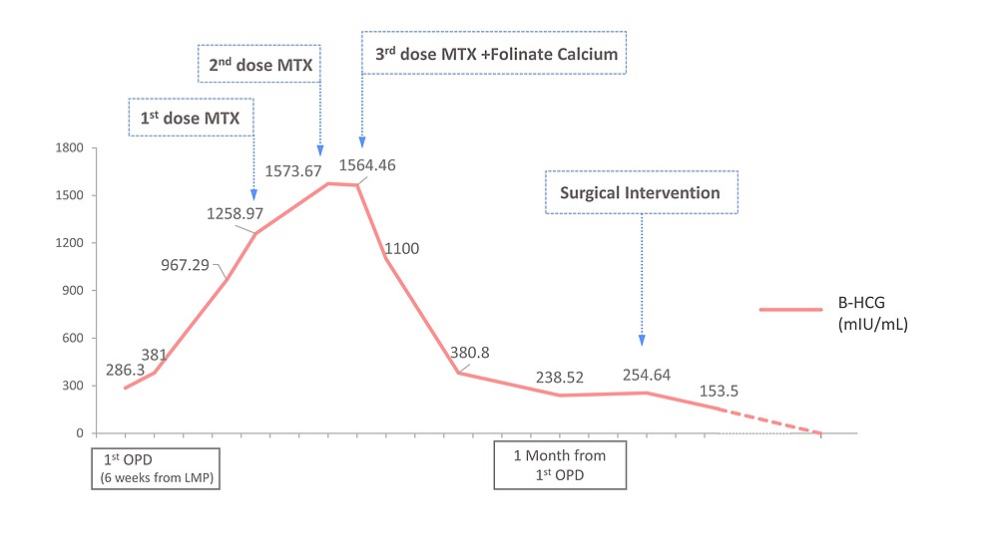
The βHCG level throughout the treatment course. The βHCG level was gradually increased since the first day the patient visited the OPD. When the level reached 1,258 mIU/mL, she received the first MTX injection (day one). On day four, her βHCG level elevated to 1,573 mIU/mL, with a 25% elevation. Then she received the second MTX injection. On day seven, the βHCG level was 1,564 mIU/mL. She received the third MTX injection because of the inadequate decreased ratio (<1%). After the three doses of MTX, the βHCG level decreased gradually and stopped decreasing after one month. βHCG: beta-human chorionic gonadotropin; MTX: methotrexate; OPD: outpatient department; LMP: last menstrual period.

Two days later, the patient’s level of βhCG had gradually elevated to 381 mIU/mL. After one week, her level of βhCG was elevated to 967.29 mIU/mL. Two more days later, TVUS showed the left adnexal mass had slightly shrunk and measured 2.3 x 1.3 cm (Figure 1B), without obvious peripheral blood flow; and the level of βhCG was 1,258.97 mIU/mL. After discussion, the patient was prescribed MTX (50 mg/m^2^ body surface area) intramuscular injection (day zero). On the fourth and seventh days after the injection, the ratios of βhCG decrease were both <15% (Figure 2). The patient was then prescribed the second and third doses of MTX. Folinate calcium also accompanied the third dose of MTX on day seven.

**Figure 3:**
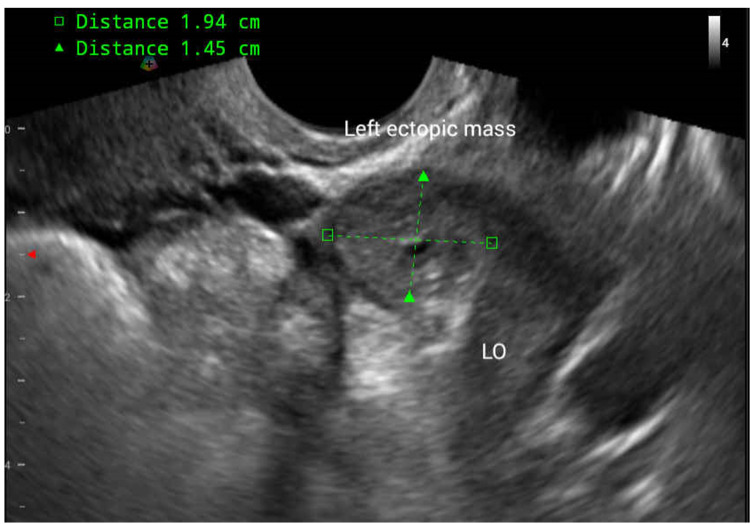
TVUS at the emergency department. Left tubal ectopic mass measuring 1.9 x 1.5 cm. Compared with the previous TVUS images, the size of the mass had shrunk after three doses of MTX. TVUS: transvaginal ultrasonography; MTX: methotrexate.

Two days after the day seven MTX injection, the patient presented to the emergency department because of sudden onset abdominal pain located at the left quadrant of her lower abdomen. On examination, she was afebrile and hemodynamically stable. A physical examination revealed mild palpable tenderness over the A7-A8 area, and a vaginal examination revealed lifting pain over the left adnexa site. Laboratory values were significant for hemoglobin (11.4 g/dL) and βhCG level (1,100 mIU/mL). TVUS showed a left adnexal mass measuring 1.9 x 1.5 cm, and no free fluid in the cul-de-sac or bilateral gutter site (Figure 3). In a relatively stable condition, she was discharged from the emergency department.

**Figure 4:**
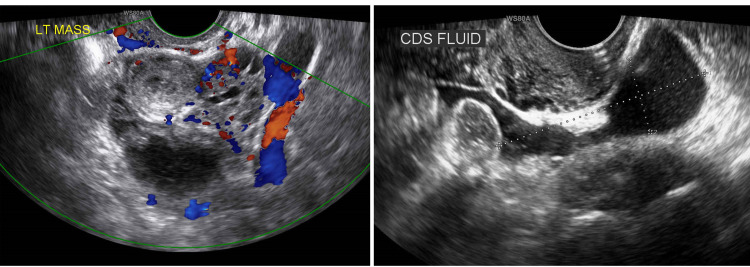
TVUS after one month from the first OPD visit. TVUS two weeks after the third MTX injection. Left tubal ectopic mass with the peripheral flow, measuring 4.9 x 4.3 x 4 cm. Some fluid accumulated in the cul-de-sac, measuring 5 x 1.7 cm. TVUS: transvaginal ultrasonography; OPD: outpatient department; MTX: methotrexate; LT: left; CDS: cul-de-sac.

On the 14th day and 21st day after the initial treatment with MTX, the levels of βhCG were decreased to an acceptable ratio. TVUS on the 21st day from initial MTX revealed enlargement of the left ectopic lesion, measuring 4.9 x 4.3 x 4 cm. It was noted that some fluid was accumulated in the cul-de-sac (Figure 4). A week later, the level of βhCG had elevated from 238.52 mIU/mL to 254.64 mIU/mL. After a full discussion, she understood the possibility of persistent EP and favored having an operation as opposed to the fourth dose of MTX. A laparoscopic procedure with salpingectomy and chromotubation under general anesthesia was arranged.

**Figure 5:**
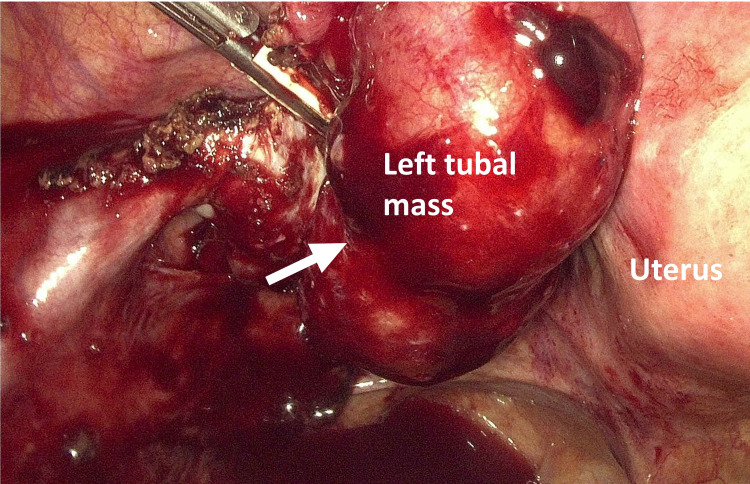
Operative findings of the laparoscopic salpingostomy. Left tubal ectopic mass measuring about 5 x 4 cm. About 200 ml of blood accumulated in the cul-de-sac.

Laparoscopic findings confirmed distention of the left adnexa with an EP mass. A small paratubal cyst next to the right fallopian tube and right hydrosalpinx was observed. About 200 ml of blood was accumulated in the cul-de-sac (Figure 5). The left tubal ectopic was removed with left salpingectomy, and a right cystectomy was performed. The right fallopian tube was tortuous with hydrosalpinx. Chromotubation was performed and the infusion of methyl blue dye only caused engorgement of the right mesosalpinx. The estimated blood loss of the surgery was <50 ml.

Histopathological reports of the resected left fallopian tube showed the presence of blood clots admixed with a few chorionic villi. These chorionic villi had a normal appearance and were covered with cytotrophoblasts and syncytiotrophoblasts; the hydropic change was noted focally. The right paratubal cyst showed a thin-walled cystic lesion lined by Mullerian epithelium. The postoperative care of the patient was uneventful, and she was discharged on the second day after surgery. Her βhCG level was <1 mIU/mL in the next month's visit.

## Discussion

Tubal EP accounts for over 90% of all classifications of EP. As the most common type of EP, it is frequently observed by clinical physicians when seeing patients in obstetrics outpatient departments for early pregnancy examinations. When the βhCG level is over 1,500 mIU/mL [10], TVUS should be performed carefully to check if there is a gestational mass inside or outside the endometrial cavity. Despite the classic triad of symptoms (amenorrhea, followed by transvaginal bleeding in the first trimester and abdominal pain), some patients with EP can be diagnosed before symptoms appear and they are still hemodynamically stable.

If the patient is stable and has no contraindications, an MTX injection is recommended as the first-line treatment option instead of surgical intervention [11,12]. Contraindications include intrauterine pregnancy, ruptured EP, hemodynamically unstable status, moderate to severe anemia, leukopenia or thrombocytopenia, renal or hepatic dysfunction, active pulmonary disease, peptic ulcer disease, hypersensitivity to MTX, or breastfeeding [12].

Treatment success is defined as a decline in serum βhCG to undetectable levels without other side effects. Conversely, treatment failure is considered when surgical intervention is needed for the clinical symptoms or serum βhCG are not declining adequately [11]. Predictors of MTX treatment failure include high initial serum βhCG level (>5,000 mIU/mL), the volume of the gestational mass (≥3.5 cm) [9], the presence of adnexal fetal cardiac activity or free peritoneal blood, and whether the βhCG was rapidly increasing (>50%/48 h) before the MTX treatment [7-9,13,14]. Before the treatment is started, these factors should be taken into consideration and discussed with the patient.

After the initial MTX treatment, serum βhCG levels should be reassessed on day three or day four. If a 15% decrease is observed, patients should be followed weekly until the βhCG level is undetectable or less than 5 mIU/mL. If the decline is not over 15% compared with the baseline level at initial treatment, the second dose of MTX should be considered [12]. Although the treatment success rate is high, at 78-96%, in selected patients [7], close follow-up is needed.

In this case, the patient seemed to be an ideal candidate for MTX treatment, since she had no contraindications for MTX and no factors that might impact the treatment result. However, her βhCG level increased after the first MTX injection, and multiple-dose treatment ultimately failed. The reasons for MTX treatment failure in patients who meet the criteria are rarely discussed.

A study by Lipscomb et al. reported that previous EP was associated with a significant increase in systemic MTX therapy failure (P = 0.0004, odds ratio 3.12), but that previous treatment method (salpingectomy, salpingostomy, or previous systemic MTX) did not have a significant effect on the likelihood of treatment failure [7,15]. In an analysis presented by Laibl et al., patients with a history of EP were nearly four times more likely to have systemic MTX treatment failure [16]. They hypothesized that there might be scarring or altered blood flow due to previous damage to the area of the fallopian tubes [16]. Some of the risk factors for EP are thought to cause damage to the fallopian tubes. For example, a history of previous tubal surgery, sexually transmitted infection, and cigarette smoking [17,18]. Previous EP also increased the risk of recurrence of EP [19], but it is hard to determine whether the previous EP is the cause or the outcome of previous tubal damage. The effect of the previous EP on the contralateral fallopian tube was not discussed either. Thus, damage to the fallopian tube might also have a negative effect on systemic MTX treatment of EP.

Although the effect of a previous EP on subsequent systemic MTX treatment failure remains uncertain, a previous study by Lipscomb et al. found that patients with a history of the previous EP tended to have poorer reproductive histories, with slightly older age, and a history of greater gravidity and lower parity [15]. These patients might already have damage to their fallopian tube, and they would be more prone to accept systemic MTX treatment, instead of other more interventive procedures. Further prospective studies are needed to clarify the consequences. However, the fact that patients with repeat EP appear more prone to systemic MTX treatment failure should be considered and discussed with the patient prior to the treatment.

Failure of systemic MTX therapy often results in tubal rupture and intra-abdominal hemorrhage. Urgent surgical intervention or transfusion can be required [10]. Patients who accepted systemic MTX had high rates of returning to the emergency department, not only for emergent conditions but also for complications such as abdominal tenderness [20]. When discussing the systemic treatment plan with the patient, the negative impact of previous EPs and the associated complications should be mentioned.

## Conclusions

Early ectopic tubal pregnancy can be managed medically with a high success rate. However, a previous history of EP indicates an increased risk of treatment failure and should be mentioned to the patient when discussing their treatment options. The likelihood of the patient visiting the emergency department is also higher in the follow-up period.
